# From support to therapy: rethinking the role of nutrition in acute graft-versus-host disease

**DOI:** 10.3389/fimmu.2023.1192084

**Published:** 2023-06-08

**Authors:** Rachel Limpert, Pan Pan, Li-Shu Wang, Xiao Chen

**Affiliations:** Division of Hematology and Oncology, Department of Medicine, Medical College of Wisconsin, Milwaukee, WI, United States

**Keywords:** graft-versus-host disease, nutrition, nutritional intervention, gut microbiota, intestinal barrier

## Abstract

Allogeneic Hematopoietic stem cell transplantation (HSCT) offers a potential cure for patients with hematologic malignancies. Unfortunately, graft-versus-host disease (GVHD) remains a major obstacle to the greater success of this treatment. Despite intensive research efforts over the past several decades, GVHD is still a major cause of morbidity and mortality in patients receiving allogeneic HSCT. The genetic disparity between donor and recipient is the primary factor that dictates the extent of alloimmune response and the severity of acute GVHD (aGVHD). However, some nongenetic factors are also actively involved in GVHD pathogenesis. Thus, identifying host factors that can be readily modified to reduce GVHD risk is of important clinical significance. We are particularly interested in the potential role of nutrition, as a nongenetic factor, in the etiology and management of aGVHD. In this article, we summarize recent findings regarding how different routes of nutritional support and various dietary factors affect aGVHD. Since diet is one of the most important factors that shape gut microbiota, we also provide evidence for a potential link between certain nutrients and gut microbiota in recipients of allogeneic HSCT. We propose a shifting role of nutrition from support to therapy in GVHD by targeting gut microbiota.

## Introduction

Allogeneic hematopoietic stem cell transplantation (HSCT) is a potentially curative treatment for patients with hematologic malignancies including leukemia, lymphoma, and multiple myeloma (1). Although the pretransplant conditioning regimen, consisting of chemotherapy with or without radiation, can kill some malignant cells directly, the curative potential of allogeneic HSCT largely depends on a graft-versus-leukemia (GVL) effect that is mediated by mature donor T cells in the allograft (2). Unfortunately, donor T cells can also play a detrimental role and afflict patients with graft-versus-host-disease (GVHD) (3, 4). Acute GVHD (aGVHD) occurs because donor T cells recognize the genetically incompatible host as foreign and mount a strong alloimmune response, resulting in multiple-organ damage of the transplant recipient. Severe aGVHD can be fatal, and it remains a major obstacle to the greater success of allogeneic HSCT (5–7).

The genetic disparity between donor and recipient is the primary factor that dictates the extent of alloimmune response and the severity of aGVHD. In clinical settings, some established GVHD risk factors also include sex-mismatched transplants, older recipient age, more intense conditioning regimens, etc. (8). These observations suggest that some nongenetic factors are actively involved in GVHD pathogenesis. Unfortunately, these factors cannot be changed within clinics to reduce GVHD risk. Therefore, identifying host factors that can be readily modified to reduce GVHD risk is of important clinical significance. We are particularly interested in the potential role of nutrition, as a nongenetic factor, in the etiology and management of aGVHD. The dietary patterns and nutritional status of allogeneic HSCT recipients vary substantially, but how these factors contribute to aGVHD risk has not been well explored. Also, diet and nutrients can be easily modified with the hope of gaining therapeutic benefits. However, the potential value of using nutritional interventions to reduce the complications of allogeneic HSCT has largely been overlooked. In this article, we summarize recent findings regarding how different routes of nutritional support and certain dietary factors affect aGVHD. We also discuss a potential link between certain nutrients and gut microbiota during aGVHD. We propose that dietary and nutritional intervention may be useful as a safe, easily applicable, and inexpensive monotherapy or adjunct therapy for aGVHD.

## Routes of nutritional support and GVHD

Nutritional status is a significant variable among patients receiving allogeneic HSCT. Prior to transplantation, patients have dietary habits and nutritional statuses that are substantially different from each other. After transplantation, they often develop macronutrient and micronutrient deficiencies secondary to changes in dietary intakes, severe mucositis, gastrointestinal GVHD, therapeutic toxicity, etc. (9, 10). Malnutrition, low levels of serum albumin, and severe weight loss have been associated with poor prognosis in patients after HSCT (11–14). These patients often cannot tolerate adequate oral intake and therefore must rely on parenteral nutrition (PN) and/or enteral nutrition (EN). However, prolonged dependence on PN can cause metabolic problems including fluid and electrolyte imbalances, hyperglycemia, and hyperlipidemia. These changes in host metabolism may have negative impacts on GVHD. For example, severe hyperglycemia post-transplant has been shown to be associated with increased risk of developing aGVHD in two clinical studies (15, 16). These observations may be partially explained by increased inflammatory cytokines associated with hyperglycemia.

Diet management practices vary across transplant centers. Despite higher costs and associated complications, PN often remains a standard route and sometimes sole option for many HSCT patients. Some studies have examined whether routes of nutritional administration affect GVHD outcomes. Three large, retrospective studies have compared clinical outcomes between EN and PN in HSCT patients. In general, these three studies observed lower non-relapse mortality and fewer incidences of severe aGVHD in patients that received EN compared to patients who received PN. One study observed this trend in pediatric patients (17), while the other two observed a similar trend in adult patients (18, 19). In addition to PN and EN, Beckerson et al. also included a group that received inadequate oral nutrition, and these patients had similar outcomes to those that received PN. It is worth mentioning that published evidence favoring EN is of retrospective nature. Also, more critically ill patients (e.g., early sepsis, severe aGVHD) tend to require PN more often. An interesting question that should be addressed in future studies is whether EN is still better if PN includes intestinal villus protection as is used in intensive care units.

Several smaller trials also indicate a beneficial trend towards EN rather than PN for better nutritional management for HSCT patients, despite limited patient number and mixed results (20–22). In these studies, patients who received EN showed improvement in a variety of parameters. Notably, Guieze et al. saw a lower rate of transfer to intensive care, and Seguy et al. observed significantly lower incidence of grade III/IV acute GVHD and decreased early 100-day mortality rate due to infection.

Given that EN is shown to be more beneficial than PN for GVHD outcomes, oral nutrition may also be beneficial for this patient group when it can be tolerated. One study applied a stepwise upgrade diet protocol to children suffering from gastrointestinal aGVHD. This dietary management appeared to contribute to a faster improvement of GI GVHD in these patients (23). A different group implemented an evidence-based nutrition support pathway for HSCT patients. As a result, these patients showed improved oral intake and relied less on PN than those patients who were not involved in a nutrition support pathway (24). Another study found that out of 228 HSCT patients, 144 were able to eat daily during their initial hospital stay, and 28 of those 144 patients did not need additional PN. The 84 patients on PN who were unable to tolerate any oral intake were divided into groups based on how many days they went with no oral intake. These researchers found a correlation between days without oral intake and grade III/IV aGVHD. Patients who went more than 9 days without oral intake had the poorest prognosis (25). Thus, oral nutrition may have important prognostic impacts in patients after allogeneic HSCT. If PN is necessary, as it often is for patients with severe aGVHD, reintroduction of oral intake as soon as it can be tolerated, even in addition to PN, may better support these patients. Of course, this should be initiated carefully and gradually with consideration of the patient’s overall condition and gut function. Improving food taste and smell may help increase oral intake. Further studies and clinical trials should attempt to optimize the protocol of transitioning patients from PN dependency to sole oral feeding cautiously and effectively.

Overall, there seems to be a beneficial trend toward EN in HSCT patients. Therefore, if total parenteral nutrition (TPN) is initiated, early reintroduction of oral intake is likely to improve clinical outcomes. The observation that GVHD was reduced due to better oral intake in patients receiving home care compared with hospital care further supports this premise (26). Atrophy of GI mucosa, weakness of gut barrier function, and lack of gut bacterial fermentation associated with sole PN support may partially explain poor patient outcomes. A comprehensive evaluation of individual patients’ nutritional status will be the foundation for optimal dietary management for this patient group (27).

## Nutritional factors and GVHD

### Amino acids

Tryptophan is an essential amino acid found in high-protein foods. It is a precursor to serotonin and melatonin and is metabolized through the kynurenine, serotonin, and indole pathways (28). The breakdown of tryptophan along the kynurenine axis is mediated by the rate-limiting enzyme indoleamine 2,3-dioxygenase (IDO). Tryptophan depletion or the accumulation of its cytotoxic metabolites can cause T-cell anergy and apoptosis, facilitating the induction of immune tolerance (29). In preclinical studies, Jasperson and colleagues found that IDO expression was increased in HSCT recipient intestinal tissues. IDO deficiency in recipient mice accelerated GVHD lethality with significantly increased colonic T cell infiltration and inflammation, demonstrating a protective role of IDO induction during intestinal GVHD (30). Further, the administration of exogenous tryptophan metabolites led to reduced GVHD (31). Dysregulated tryptophan metabolism in the brain was also evident in mouse models of GVHD (32). Human studies confirmed the involvement of IDO in regulating intestinal GVHD (33). In addition, tryptophan catabolism was induced in patients after allogeneic HSCT, and kynurenine levels were associated with GVHD severity (34). Tryptophan can also be utilized by gut bacteria (28). Bacterial catabolism of tryptophan produces indoles that improve intestinal barrier function and gut health. Importantly, recent studies have shown indole and its derivatives can improve gut barrier integrity and protect against GVHD in mouse models (35).

Glutamine is a non-essential amino acid but serves as a preferable nutrient for enterocytes, hepatocytes, lymphocytes, and macrophages. Glutamine deficiency can lead to increased gut permeability and higher secretion of inflammatory cytokines (36). Interestingly, it has been reported that oral glutamine supplementation improves intestinal barrier function and reduces GVHD-associated tissue damage in animal models (37). The idea of supplementing glutamine to HSCT patients has been proposed and investigated (38). One meta-analysis included 17 randomized controlled trials of HSCT patients with supplemental glutamine (39). Most of these studies were small and varied in terms of recipient age, transplant type (autologous or allogeneic), route of administration (oral or intravenous), and administration schedule. Overall, there was no effect of oral or intravenous glutamine on transplant-related mortality at day 100. Oral glutamine might reduce mucositis in HSCT recipients. Intravenous glutamine might suppress infection but increase the risk of relapse according to two small studies (39). However, these observations should be interpreted cautiously since they were mostly based on a few small trials. Large, well-designed trials may provide insight into whether glutamine can improve GVHD.

### Dietary fat

Allogeneic HSCT is associated with significant changes in host lipid metabolism (40). These changes can result in dyslipidemia which is often observed in transplant recipients (40). There is evidence that obesity is associated with worse clinical outcomes in patients receiving allogeneic HSCT (41, 42). A trend toward increased risk of grade II-IV aGVHD with increased body mass index (BMI) has been reported (43). Recent studies demonstrated that obesity causes changes in host gut microbiota and augments GVHD after allogeneic HSCT (44, 45).

Statins are lipid-lowering drugs frequently used to treat high serum cholesterol, but their potential immunomodulatory effect has been studied in the context of aGVHD (46–48). In one retrospective study, pravastin was used for endothelial protection and reduced non-relapse mortality after HSCT, though not due to a lower incidence of aGVHD. However, patients who did suffer from aGVHD benefited more from statin treatment than patients without aGVHD (49). A murine study found that atorvastatin reduced aGVHD lethality without affecting GVL effect when both donor and recipient were dosed with the drug. This result was attributed to the drug’s effect on the polarization of donor T cells and the function of host antigen presenting cells (APCs) (50). Another group observed similar results when testing the effects of simvastatin on human APCs *in vitro* (51). These results also indicate the involvement of lipids in GVHD pathogenesis. In addition to the immunomodulatory effects, statins lower lipid and cholesterol levels. Fatty acid metabolism is implicated in the clonal expansion and function of donor alloreactive T-cells (52–54). Use of statins may help reduce the negative effect that a high-fat and high-cholesterol diet potentially has on HSCT patients. Lipidomic studies using patient blood samples have shown that pretransplant lipid signature has the potential to be used as a biomarker of GVHD (55).

Essential fatty acids (EFAs) are polyunsaturated fatty acids (PUFAs) that cannot be synthesized by the body and must be obtained from diet (56). There are two classes of EFAs: omega-6 and omega-3. Each plays a different role in regulating inflammation. omega-6 PUFAs promote inflammation by producing proinflammatory cytokines while omega-3 PUFAs are generally considered to have anti-inflammatory properties. The potential effects of EFAs on GVHD have been explored in a B6→Balb/c mouse model of allogeneic HSCT with a chemotherapy-based conditioning regimen. Interestingly, omega-3-rich fish oil supplementation worsens GVHD by reducing the reconstitution of regulatory T cells (57). The precise role of dietary fatty acids in GVHD warrants further investigation.

### Vitamins

Vitamin D is a steroid hormone known to exert an anti-inflammatory function and promote immune tolerance (58, 59). The active form of vitamin D is 1,25-dihydroxyvitamin D3 [1,25(OH)_2_D_3_] (60). Vitamin D interacts with vitamin D receptors (VDRs) expressed in various immune and nonimmune cells. Given the broad and critical role of vitamin D in the immune system, many studies have investigated the relationship between vitamin D status and clinical outcomes in HSCT patients (61–64).

Lower serum vitamin D levels were reported before and/or after HSCT in nearly all studies, despite different thresholds to define vitamin D insufficiency/deficiency (65–75). Some studies found significant associations between vitamin D insufficiency/deficiency with decreased survival (65, 70, 72, 74) or higher incidence of chronic GVHD (69, 72), while other studies observed no significant associations. Interestingly, one study reported a more frequent incidence of acute GVHD among patients with sufficient vitamin D levels (70). The inconsistency between studies may be attributed to different patient populations, various cut-off levels for vitamin D deficiency, different HSCT conditioning regimens, and lack of information on VDR expression levels etc. A prospective multi-center phase I/II clinical trial observed that vitamin D supplementation was associated with a significantly lower incidence of chronic GVHD at one year after transplantation (76). Further studies showed that patient VDR polymorphism contributes to the outcomes of vitamin D supplementation (77). Overall, vitamin D insufficiency/deficiency is quite common in patients undergoing HSCT. Whether vitamin D levels are associated with GVHD risk and whether vitamin D supplementation improves transplant-associated complications such as aGVHD remain inconclusive. Defining the best serum marker for vitamin D levels, standardizing cut-off levels of vitamin D deficiency, and identifying which group of patients are most likely to benefit from vitamin D supplementation will advance research in this area.

Vitamin A is another fat-soluble vitamin that has been implicated in GVHD pathogenesis (78, 79). Retinoic acid (RA), the active metabolite of vitamin A, has a broad range of effects on the immune system. RA binds to retinoic acid receptors (RARs), and either an immunosuppressive or a proinflammatory response is observed depending on the context of RA/RAR signaling. Whether vitamin A levels affect aGVHD risk remains unclear. Some clinical studies reported negative impacts of lower serum vitamin A levels on GVHD severity in pediatric patients, suggesting potential benefits of supplementing vitamin A to these patients (80, 81). However, it has also been reported that pre-transplant serum vitamin A levels do not appear to affect the development of acute GVHD in adult patients undergoing allogeneic HSCT (82). Importantly, a population of RA-responsive pathogenic donor CD8^+^ T cells has been identified in patients with gastrointestinal GVHD (83).

Preclinical studies examining the relationship between RA signaling and GVHD also reported mixed results (84–88). Yang et al. observed a protective effect of RA against GVHD in a B6 → B6D2F1 mouse model of aGVHD (84). By contrast, our group and others showed that enhancing RA/RAR signaling by exogenous RA exacerbated the development of gastrointestinal GVHD (85–88). Furthermore, our recent studies showed that enhancing RA signaling may negatively impact gut barrier integrity during GVHD (89). Given the pleiotropic effects of RA on immune and nonimmune cells as well as its highly context-dependent effects, it is not surprising that different and sometimes contradictory results are found in the literature. Overall, both preclinical and clinical evidence indicates an important role of RA/RAR signaling in the development of gastrointestinal GVHD. Serum vitamin A levels may provide some useful information, but the expression of RARs or RA levels in tissue sites such as the gut seem more valuable to define their relation to GVHD outcomes. Such information may be obtained from tissue biopsy specimens obtained when diagnosing and staging gastrointestinal GVHD. Further studies are needed to determine how to modify RA signaling or use vitamin A advantageously for GVHD patients.

### Choline

Some dietary and nutritional factors have the potential to worsen GVHD. A recent study showed that a choline-rich diet accelerated GVHD-associated mortality in mice (90). Choline is an essential nutrient for humans and is required in many biological processes including the formation of cell membranes and the synthesis of neurotransmitters (91). Choline can also be utilized by intestinal bacteria, producing the toxic metabolite Trimethylamine N-oxide (TMAO) which can cause vascular inflammation (92). Wu et al. found that choline-derived or exogenously supplied TMAO aggravated experimental GVHD. Mechanistic studies revealed that TMAO enhanced M1 macrophage polarization *via* NLRP3 inflammasome activation. A high-choline diet significantly increased serum TMAO levels and resulted in more severe GVHD target organ damage (90). This study highlighted the detrimental effects of some dietary components on GVHD that are often unrecognized by clinicians.

## Gut microbiota and GVHD

Our gut microbial communities contain up to 1000 bacterial species and have immense impact on human metabolism, nutrition, and immune function (93). Disruption of gut microbiota has been linked to many diseases including cancer, obesity, diabetes, malnutrition, and inflammatory bowel disease (94–98). Perturbation of gut microbiota is commonly observed in recipients of allogeneic HSCT (99–103). Changes in the diversity and composition of gut microbiota can be due to several factors including conditioning-induced gut damage, changes in diet, prophylactic and/or therapeutic use of antibiotics, and inflammation caused by GVHD (101, 103–105). Patients with steroid-refractory aGVHD have a poor prognosis and this detrimental complication is associated with endothelial dysfunction. Early sepsis is an endothelial complication that can already be predicated prior to transplantation (106–109), which often results in significantly higher antibiotic exposure of these patients. It is thus conceivable that endothelial dysfunction can influence the gut microbiome by higher incidence of sepsis.

In the gut microbiota of allogenic HSCT patients, there is often a loss of microbial diversity, dominance of a single bacterial species, and the expansion of opportunistic pathogens (110–113). Studies found that patients with more diverse gut microbiota had better survival rates and lower non-relapse mortality (111, 112). The increased abundance of certain bacterial species, especially *Blautia*, was associated with reduced GVHD mortality (110). Antibiotics with broad-spectrum effects appear to correlate with higher GVHD-related mortality (114). Mechanistically, antibiotics such as carbapenem can expand mucus-degrading bacteria that impair gut barrier function and aggravate aGVHD (115). Thus, the antibiotics used in this patient group should be carefully chosen. It is clear from these studies that certain bacteria are beneficial within the GI tract of HSCT patients while others could be harmful. Higher diversity of gut microbiota and enrichment of beneficial bacteria are crucial for better GVHD outcomes.

Given this well-demonstrated trend, strategies that aim at preserving diversity and potentially enhancing the richness of beneficial gut microbiota have been proposed and investigated. One approach is the introduction of live microbes through fecal microbiota transplant (FMT). Pilot trials have shown that successful FMT from patients’ spouses, relatives, or even random, healthy donors led to partial or complete resolution of gastrointestinal GVHD (116, 117). Subsequent reports provided further evidence that FMT improved gut microbiota diversity and composition in GVHD patients (118, 119). Despite these positive results, the potential risk of bacteremia induced by FMT needs to be further addressed before this approach can be used more widely in this patient group (120). Probiotics may also be used to introduce live microbes. The effects of probiotics have been tested in animal models of HSCT. Oral administration of *Lactobacillus rhamnosus* GG led to improved post-transplant overall survival and reduced experimental GVHD (121). A more recent study demonstrated that a single strain of *Bacteroides fragilis* can improve gut barrier integrity and mitigates GVHD in mice (122). In human studies, the use of probiotics appeared to be safe in HSCT patients, but whether they improve transplant outcomes remains to be determined (123, 124).

Nutrients that are considered prebiotics and postbiotics have also been investigated. Prebiotics stimulate the growth of beneficial bacteria in the gut. Unlike probiotics, these are nutrients found in food and supplements and do not introduce live microbes. Some commercially available prebiotics include inulin, galacto-oligosaccharides (GOS), and fructo-oligosaccharides. Fructo-oligosaccharides are well-tolerated when given to patients receiving allogeneic HSCT and can modify gut microbiota composition and phenotypes of some immune cells (125). Yoshifuji et al. showed that the combination of resistant starch and other dietary prebiotics effectively reduced mucosal damage after transplantation, preserved microbiota diversity, and mitigated GVHD (126). Patients with higher baseline gut microbiota diversity appeared to benefit most from prebiotic supplementation, likely because fermentation of prebiotics requires a relatively intact gut microbiota. Holmes et al. recently found that mice supplemented with GOS had improved post-transplant survival in a well-established murine GVHD model, but only when control and experimental mice were also treated with antibiotics. This suggests that prebiotics may mitigate injury to the gut microbiome caused by antibiotics (127).

Given the potential infection risk associated with probiotics in immunocompromised HSCT patients and the requirement of a healthy gut microbial community to digest prebiotics, postbiotics seem to be a safer and more effective strategy (103, 128). Postbiotics are metabolites of beneficial gut bacteria. The most promising and well-studied postbiotic is butyrate, a short-chain fatty acid that can promote gut health. Indeed, it has been demonstrated that butyrate can mitigate experimental gastrointestinal GVHD (129).

## Interplay between nutritional factors and gut microbiota during GVHD

Diet is one major factor that shapes gut microbiota in humans and experimental animals (130). Dietary macronutrients and micronutrients can influence the diversity and composition of gut microbiota. It has been well established that a high-fat, high-animal protein, and low-fiber dietary pattern contributes to the development of many metabolic and inflammatory diseases such as obesity, cancer, and cardiovascular diseases (131). Gut dysbiosis is often observed in these patients and is linked to their eating habits.

Accumulating evidence supports the active interplay between nutrients and gut microbiota during GVHD. We have already discussed the interaction of tryptophan, choline, and prebiotics with gut microbiota during GVHD. There is also evidence that tyrosine, a nonessential amino acid, impacts gut microbiota. In a mouse model of allogeneic HSCT, tyrosine was found to be reduced in the gut of GVHD mice (132). This was accompanied by a dramatic reduction of tyrosine metabolites as revealed by metabolomic analyses. Recipient mice fed a tyrosine-supplemented diet showed prolonged survival and decreased tissue damage in the early stages of GVHD. Tyrosine supplementation led to changes in the composition of gut microbiota and host metabolism, demonstrating a crosstalk between this amino acid and gut microbiota during GVHD (132).

There is also ample evidence that vitamins, particularly fat-soluble vitamins A and D, can actively participate in regulating gut microbiota (133–136). In the setting of allogeneic HSCT, we recently showed that vitamin A can modulate gut microbiota during GVHD (89). Vitamin A-supplemented mice had a reduced gut microbiota diversity. The abundance of opportunistic pathogens such as *Escherichia-Shigella* was increased in these mice, but the abundance of *Bacteroides* and *Lactobacillus*, two beneficial bacteria, were significantly decreased. In our model, vitamin A supplementation is associated with a higher degree of gut dysbiosis and inferior transplant outcomes.

Lactose, a nutrient commonly found in dairy products, has been shown to be critically involved in GVHD development (137). Lactose is required for the expansion of *Enterococcus* which can intensify gut inflammation. A lactose-free diet reduced the abundance of *Enterococcus* and mitigated GVHD severity in mice. Fecal domination by *Enterococcus* early after transplantation predicts a reduced overall survival and more severe GVHD. This study provides direct evidence of a causal relationship linking a common nutrient, gut microbiota, and the severity of intestinal and systemic alloimmune responses. Many transplant centers now provide a lactose-free diet to patients with intestinal GVHD (138).

Modification of gut microbiota is likely to be an important mechanism by which nutritional factors affect GVHD development, as being primarily discussed in this article. However, certain nutrients and/or their metabolites can also directly act on immune or nonimmune cells that are critically involved in the pathogenesis of aGVHD. For example, vitamins A and D can have pleiotropic effects on donor T cells and host APCs/DCs to modulate GVHD risk as reviewed previously (62).

## Discussion and future directions

Despite the limitations that GVHD exhibits on the success of allogeneic HSCT, there are currently few effective options for prevention and treatment of the disease. Accumulating evidence points to nutrition as an important and modifiable nongenetic factor that may influence GVHD risk. A nutritional approach may be beneficial as dietary changes and nutritional supplements can be quickly and easily implemented by physicians into patient care plans.

As outlined in the above sections, enteral nutrition provides nutritional support superior to parenteral nutrition for patients with GVHD. Accumulating evidence also indicates that certain nutrients confer protective effects while other nutrients can worsen GVHD. Tryptophan and tyrosine supplementation appear to lead to better GVHD outcomes in animal models, but more human studies are needed to confirm these results. Nutrients that are considered essential for the general population could sometimes be detrimental for recipients of allogeneic HSCT. For example, choline accelerated GVHD mortality in one murine study. Vitamin A appears to have mixed results in animal studies. Due to its known influence on the immune system, vitamin A deserves to be studied further in the context of aGVHD. Pre-transplant nutritional status should be carefully considered and assessed for any potential indicators of GVHD risk.

From a therapeutic standpoint, we propose that nutritional intervention may be an attractive option for patients with GVHD. It is encouraging that various dietary intervention trials are ongoing (**Table 1**). There is convincing evidence indicating that gut microbiota diversity is directly related to the severity of gastrointestinal GVHD. Thus, interventions that can preserve intact diversity and restore injured gut microbiota should be investigated. Fecal microbiota transplant and probiotics are two options that deserve further exploration. There is also a clear interplay between the status of gut microbiota and dietary habits. This is true for all populations but is especially crucial in the context of allogeneic HSCT. The combination of fecal microbiota transplant and dietary intervention should be considered in clinical trials. Prebiotics and certain nutrients may also be used to restore microbiota diversity and composition to reduce GVHD. Unlike drug-based therapeutics that are expensive and require lengthy approval processes, nutrition-based approaches are expected to be inexpensive and easily applicable in the clinic with good safety profile. However, it is important to keep in mind that the translation of findings in animal models into clinical settings isn’t always straightforward and cannot be taken for granted. Also, severe aGVHD is a catabolic catastrophe that is unlikely to be managed by pure nutritional intervention. Instead, nutritional intervention can be easily incorporated into existing GVHD prevention or treatment protocols to improve patient outcomes. By adding beneficial nutritional and/or dietary factors, the use of drugs with significant side effects, such as steroids, may be reduced.

**Table 1:** Some human clinical trials of dietary and nutritional interventions for managing GVHD in HSCT patients.

Trial title	Trial number	Phase	Population	Participant number	Interventions and Observations	Trial Status	Trial Location
Therapeutic Use of Intravenous Vitamin C in Allogeneic Stem Cell Transplant Recipients	NCT03613727	II	18-77 years old HSCT patients	60	IV vitamin C 50 mg/kg/day from day +1 to day +14, then oral vitamin C 500 mg twice each day from day +15 to day +180	Recruiting	Virginia Commonwealth University, United States
Study on Intelligent Nutrition Support Therapy for Hematopoietic Stem Cell Transplantation Recipients	NCT05590091	Not Applicable (N/A)	12-55 years old HSCT patients	106	Intelligent nutrition support therapy for 100 days after transplantation	Not yet recruiting	Hebei Yanda Ludaopei Hospital, China
IRENE-G Study: Impact of Resistance Exercise and Nutritional Endorsement on GvHD Symptoms	NCT05111834	N/A	18 years and older HSCT patients	112	Nutritional endorsement/therapy based on individual needs and supervised resistance trainining 2x per week	Recruiting	University Hospital Heidelberg, Germany
Fecal Microbiota Transplant and Dietary Fiber Supplementation for the Treatment of Gut Graft Versus Host Disease	NCT05067595	I	18 years and older HSCT patients	72	Partcipants are randomized into four arms, all recieve FMT, two arms receive additional fiber supplementation	Not yet recruiting	Fred Hutchinson Cancer Center, United States
Nutrition and Outcomes of Hematopoietic Cell Transplantation (HCT)	NCT03419078	N/A	17 years and older HSCT patients	484	Retrospective case-note review of broad adequacy of nutritional intakes comparing enteral nutrition and parenteral nutrition	Completed	Imperial College Healthcare, United Kingdom
A Randomized Phase IV Control Trial of Single High Dose Oral Vitamin D3 in Pediatric Patients Undergoing HSCT	NCT03176849	IV	1 to 25 years old HSCT patients	49	One time oral dose of vitamin D3 based on age and initial vitamin D level given pre-transplant	Completed	Phoenix Children's Hospital, United States
Immunomodulatory Effect of Vitamin D in Allogenic Post-transplant	NCT02600988	I/II	18 years and older HSCT patients	150	Vitamin D supplementation in three arms: Control, 1000IU/day, and 5000IU/day	Completed	Andalusian Public Foundation for Research Management in Seville, Spain
Glutamine in Treating Side Effects in Children Who Are Undergoing Stem Cell Transplantation	NCT00003898	II	1 to 21 years old HSCT patients	120	Glutamine or placebo oral supplementation day 0 to +28 or hospital discharge	Completed	Rosewell Park Cancer Institue, United States
Dietary Manipulation of the Microbiome-metabolomic Axis for Mitigating GVHD in Allo HCT Patients	NCT02763033	II	10 years and older HSCT patients	70	20g of potato starch or placebo twice daily consumed orally from day -7 to +100	Recruiting	University of Michigan Rogel Cancer Center, United States
Prebiotic Galacto-oligosaccharide and Acute GVHD	NCT04373057	I/II	18-80 years old HSCT patients	128	GOS administered at determined dose levels per protocol once daily from about 30 days before transplant to about 4 weeks after transplant.	Recruiting	Duke University, United States
Topical Vitamin D in Acute Graft Versus Host Disease of the Skin	NCT03093805	N/A	3 months to 35 years old HSCT patients	10	Calcipotriene used as the exclusive topical therapy in addition to standard of care GVHD treatment prescribed for the study subject by the primary physician	Completed	Cincinnati Children's Hospital Medical Center Cincinnati, Ohio, United States
High Dose Vitamin A in Preventing Gastrointestinal GVHD in Participants Undergoing Donor Stem Cell Transplant	NCT03719092	N/A	18 years and older HSCT patients	34	Participants receive vitamin A compound orally (PO) or enterally once prior to stem cell transplant	Recruiting	Ohio State University Comprehensive Cancer Center Columbus, Ohio, United States

N/A, Not Available.

**Figure 1:**
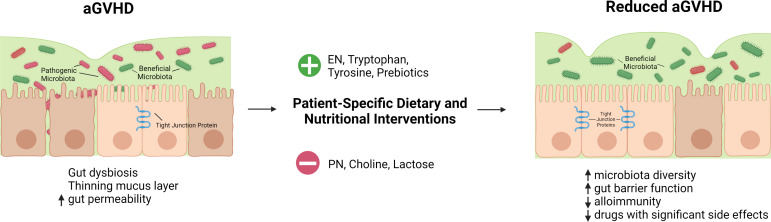
Hypothetical summary of nutritional and dietary interventions that may help reduce aGVHD. HSCT conditioning regimen, reduced oral intake, and transplant-associated inflammation lead to a thinning mucus layer, gut dysbiosis, and increased gut permeability. Gut dysbiosis is characterized by a reduction in the diversity of gut microbiota and short chain fatty acid-producing beneficial microbes as well as an expansion of pathogenic bacteria. Dietary and nutritional interventions tailored to patients' specific needs can improve these outcomes. Enteral nutrition is favorable over parenteral nutrition for patients after allogeneic HSCT. Tryptophan, and tyrosine supplementation as well as the use of prebiotics may have a beneficial effect while choline and lactose may have a negative impact. Nutritional interventions may correct gut dysbiosis, restore gut barrier function, and mitigate alloimmune response with the net outcome of reduced GVHD. 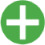-supply 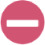-avoid. Created with BioRender.com.

In summary, nutritional management should be an integral part of medical care for patients undergoing allogeneic HSCT. Carefully designed and well-formulated nutritional support will not only improve the overall wellbeing of these patients but help mitigate transplant complications in this medically fragile patient group. In this regard, the role of nutrition could be shifting from support to therapy. There are numerous vitamins, minerals, amino acids, and other nutritional factors that impact human health and have immunomodulatory effects, many of which have not been explored in the specific context of allogeneic HSCT and GVHD. The current evidence proves that this is an area worth exploring further. There could be one or several nutritional components that are severely affecting GVHD patients, both positively and negatively (**Table 2**, **Figure 1**). Identifying these elements will help improve evidence-based guidelines for nutrition before and after transplantation. More well-designed clinical trials will help to determine potential links between nutrition and aGVHD in humans. Nutritional assessment as a standard before HSCT can also reveal how pretransplant nutritional status affects aGVHD pathogenesis. Gut microbiota is likely to serve as an important nexus linking nutrition and aGVHD outcomes during nutritional intervention. Further, in an era of precision medicine, tailored dietary intervention based on the unique nutritional status and microbial composition of individual patients is expected to achieve optimal results. We believe that a better understanding of the complex interplay between nutritional factors, gut microbiota, and mucosal immune responses will shed new light on aGVHD pathogenesis and management. We propose the use of dietary and nutritional intervention as a safe, easily applicable, and inexpensive monotherapy or adjunct therapy for patients with aGVHD by targeting gut microbiota.

**Table 2:** Summary of nutrients discussed and their potential effects on Agvhd.

Nutrient	Effect on GVHD Outcomes	Study Type	Details	References
Tryptophan	Positive	Pre-clinical	IDO deficiency accelerated GVHD lethality. Exogenous metabolites reduced GVHD. Indoles protect against GVHD.	(30, 31, 35)
Tyrosine	Positive	Pre-clinical	Mice fed a tyrosine-supplemented diet showed prolonged survival and reduced tissue damage in one study. Tyrosine led to changes in gut microbiota composition.	(132)
Glutamine	Unclear	Pre-clinical and clinical	Glutamine improves intestinal barrier function but needs further study in the context of GVHD as current trials are small and limited.	(37–39)
Lipids	Negative/Unclear	Pre-clinical and clinical	Lipid-lowering drugs reduced GVHD. Fatty acid metabolism is implicated in clonal expansion and effector function of donor T cells. Increased risk of grade II-IV acute GVHD is associated with increased body mass index (BMI). Omega-3 fish oil worsened GVHD in one study.	(43, 49–54, 57)
Vitamin D	Unclear	Pre-clinical and clinical	Some studies associated vitamin D insuffiency/deficiency with decreased survival while others found no association. One study found higher incidence of acute GVHD in vitamin sufficient patients.	(61, 64, 65, 68–70, 72–74, 76)
Vitamin A	Unclear	Pre-clinical and clinical	Some clinical studies reported negative impacts of lower serum vitamin A levels on GVHD severity in pediatric patients, but it has also been reported that pre-transplant serum vitamin A levels do not appear to affect the development of aGVHD in adult patients. Preclinical studies regarding the effects of RA on GVHD also yielded mixed results.	(80–89)
Choline	Negative	Pre-clinical	A choline-rich diet accelerated GVHD-associated mortality and increased serum TMAO levels.	(90)
Lactose	Negative	Pre-clinical and clinical	A lactose-free diet reduced the abundance of *Enterococcus* and mitigated GVHD severity in mice. A lactose-free diet is commonly recommended for patients after HSCT.	(137, 138)
GOS	Positive	Pre-clinical	Mice supplemented with GOS had improved post-transplant survival when also treated with antibiotics.	(127)
FOS	Positive	Clinical	. Fructo-oligosaccharides are well-tolerated when given to HSCT patients and can modify gut microbiota composition and phenotypes of some immune cells.	(125)
Resistant Starch	Positive	Clinical	Resistant starch combined with other prebiotics reduced mucosal damage after transplantation, preserved microbiota diversity, and mitigated GVHD in one study.	(126)

## Author contributions

RL wrote and edited the manuscript. PP wrote the manuscript. L-SW discussed the work. XC conceived the concept and topic, supervised the work, wrote, and edited the manuscript. All authors contributed to the article and approved the submitted version.
